# Drug dissolution problems in ostomy patients: a chart review and patient interview analysis

**DOI:** 10.1007/s00384-025-04907-9

**Published:** 2025-05-10

**Authors:** Yvonne Remane, Maria Astrid Stolz, Katrin Heinitz, Albrecht Hoffmeister, Matthias Mehdorn, Stefan Niebisch, Kirsten Lincke, Annett Huke, Daniel Seehofer, Thilo Bertsche

**Affiliations:** 1 Pharmacy, Leipzig University Medical Center and Medical Faculty, Leipzig, Germany; 2 Drug Safety Center, Leipzig University and Leipzig University Medical Center, Bruederstraße 32, 04103 Leipzig, Germany; 3 Medical Department II – Gastroenterology, Hepatology, Infectious Diseases, Pulmonology, Leipzig University Medical Center, Leipzig, Germany; 4 Department of Visceral, Transplantation, Thoracic and Vascular Surgery, Leipzig University Medical Center, Leipzig, Germany; 5 Clinical Pharmacy, Institute of Pharmacy, Medical Faculty, Leipzig University, Leipzig, Germany

**Keywords:** Ostomy, Gastrointestinal tract, Drug absorption, Pharmaceutical formulation

## Abstract

**Purpose:**

The pharmacokinetic properties of peroral drugs, e.g. considering dissolution, release and absorption, can be influenced by a shortened transit time after ostomy placement. Its aim was to find unexpected undissolved drugs in stoma bags and establish a correlation with formulations using patient interviews and chart review analysis.

**Methods:**

(i) A patient chart review was performed to assess prescribed drugs on the basis of their formulation and potential influence on pharmacokinetics. (ii) Subsequent to the chart review, the patient was interviewed to ascertain (ii.a) the appearance of indigested drugs in the stoma bag, (ii.b) observed changes in drug efficacy and therapy, and (ii.c) information status regarding indigestible and undissolved formulations.

**Results:**

(i) 22.4% (134/598) of the prescribed formulations were assessed as critical concerning their pharmacokinetic release characteristics. (ii.a) A total of 22.0% (11/50) of the interviewed patients indicated that they had experienced this at least once. (ii.b) Of the patients, 14.0% (7/50) observed changes in drug efficacy, and 12% (6/50) experienced changes in their therapy regimens. (ii.c) More than 60% of the patients lacked information that indigestible and undissolved formulations could be found in their stoma bag.

**Conclusions:**

A considerable proportion of formulations investigated caused problems for patients with ostomy. Additional patient education and resources are needed to support ostomy patients by managing drug release-related problems.

**Supplementary Information:**

The online version contains supplementary material available at 10.1007/s00384-025-04907-9.

## Introduction

Structured research on the pharmacokinetic properties (dissolution, release, and absorption) of peroral drugs in patients with gastrointestinal disorders is limited due to neglect [1, 2]. Surgical procedures can significantly impair bowel function, affecting basic gut functions like nutrient absorption, immune system maintenance and motility [3]. Patients with a colostomy have fewer restrictions on absorption than patients with an ileostomy [4, 5]. In addition, the transit time of the intestine is shortened, which can lead to a reduction in the time required for the absorption of nutrients and medicines [4]. Therefore, it is of particular importance to consider the physiological conditions in the gastrointestinal tract and, as a consequence, the choice of pharmaceutical formulations used in patients with an ostomy [6]. The galenic properties of a formulation influences its disintegration time and thus, represents a major factor on the liberation of its active ingredients. Formulations with slow disintegration or sustained release may present a particular challenge for patients with an ostomy [4]. Consequently, a reduced absorbed quantity of a drug results in a decrease in peroral bioavailability and a reduction in efficacy or even an absence or decrease of the therapeutic effect [7]. A comprehensive analysis of pharmaceutical formulations (galenic) used in ostomy patients, based on an evaluation of the absorption characteristics that determine release, liberation and absorption in ileostomy patients, was provided in a study by van der Linde et al. [8, 9]. However, in patients with a shortened intestine, modified or sustained release formulations may be ineffective due to rapid transit, leading to incomplete drug liberation, insufficient absorption, and premature elimination. When considering formulations, paying specific attention to the distinctive characteristics of gastroretentive formulations (i.e. with a potential prolongation of the gastric retention time) and drugs that affect the colon is of particular clinical relevance [8, 10, 11]. However, the presence of a drug in the stoma bag does not necessarily indicate a change in its efficacy. Specific pharmaceutical formulations, such as sustained-release formulations or matrix systems, are indigestible, yet the active ingredient is fully released. Some information on indigestible formulations is available in specialist information leaflets and the literature [12]. It may be hypothesised that drug liberation and, consequently, the absorption and therapeutic effect might be impaired due to the accelerated transit in ostomy patients. Nevertheless, a significant gap can be observed in literature with respect to the influence on drug therapy in ostomy. Therefore, the aim of this study was to identify unexpected undissolved drugs in stoma bags and to establish a relation with pharmaceutical formulations. First part, (i) a review of patient charts was performed to characterize the prescribed drugs in accordance with its pharmaceutical formulation and potential impact on pharmacokinetic processes. The subsequent patient interview (ii) should investigate, from the patient’s point of view, whether (ii.a) undissolved drugs were found in their stoma bag, (ii.b) this had an influence on therapeutic drug effect and their therapy regimen, and (ii.c) the extent to which the patient was prepared for this issue in advance.

## Materials and methods

### Setting and study design

The study was designed and reported in accordance with the STROBE criteria for case–control studies, as these were deemed appropriate for the study protocol. It was performed between June and September 2021 at a German University Hospital. All in- and outpatients who had previously undergone an ostomy and attended the 'lower gastrointestinal tract' consultation during the specified period were included in the study. In the context of a pilot study, consecutive sampling was employed to assess the frequency of occurrence. Therefore, a formal sample size calculation was not performed. The data were collected from a current electronic health record, a medication chart or a physician’s letter issued after January 1st 2020. The status quo of medication was recorded, without any pharmaceutical intervention on the drugs currently prescribed.

### Inclusion and exclusion criteria

Inclusion criteria were to be at least 18 years of age and, to have a criterion for interviews was to be fluent in German. Patients were enrolled after providing written informed consent for both the patient chart review and the patient interview.

Patients who underwent stoma reconstruction for up to two weeks postoperatively were excluded from the interviews. The rationale for this was twofold: first, the postoperative period was too short for patients to have had sufficient time to acclimatize to the novel therapeutic situation; second, patients were not yet familiar with the complications that could arise from the change in drug release.

### Patient chart review (i)

The electronic patient file, which contained a variety of documents, including physician letters and surgical reports, served as the basis for the patient chart review. The following items were recorded: internal patient number, date of birth, sex, disease and subsequent diagnosis for stoma insertion, location of the stoma (jejunostomy, ileostomy or colostomy), type of stoma: terminal or loop ostomy, interval after stoma placement in months, resections (if known in centimetres), anastomoses including gastric bypass operations (e.g., Roux-en-Y), diagnosis of short bowel syndrome, current prescribed drugs. All peroral drugs and pharmaceutical formulations, including buccal and sublingual formulations, were systematically documented for all enrolled patients. To characterize the prescriptions, the following items were collected: drug, brand, active ingredient, dosage, pharmaceutical formulation, and ATC code. The drugs were classified according to the first level of the ATC classification system.

### Patient interview (ii)

The patient interviews were semi-structured through a questionnaire that was based on a recently published online survey [8]. This questionnaire was developed to elicit specific information for the purpose of quantitative evaluation. The design of a semi-structured interview allows further questioning to ensure that all relevant aspects are considered. However, the possible answers were ever given in accordance to the questionnaire. In advance, the questionnaire was evaluated with an expert panel consisting of pharmacists, physicians, and stoma care nurses. The following items were documented: internal patient number (to double-check with chart analysis), type of stoma, patient-reported drug release-related problems (e.g., undissolved drugs in their bag), recording and identification of undissolved drugs (including brand, active ingredient, dosage, pharmaceutical formulation) subjective changes in therapy efficacy (after stoma insertion or drug observation), and patients’ education of the impact in therapeutic drug effect.

### Classification of drugs according to their formulation

Formulations were classified as part of a subgroup analysis. The classification system used differentiates between formulations as follows [10]: noncritical (accelerated drug release, the drug is dissolved or absorbed bypassing the intestinal tract), potentially critical (normal drug release) and critical (modified/sustained drug release or indigestible preparations) (Table 1). The classification system was applied to all formulations during the data collection process.

**Table 1:** Classification system including drug release and characteristics of the pharmaceutical formulation according to van der Linde et al. [13]

Classification	Drug Release	Pharmaceutical formulations
Noncritical	Accelerated release – drug is dissolved/absorption bypassing the intestinal tract	Solid: buccal tablets, chewable tablets or capsules, effervescent tablets, granules, lozenge, melting, powder (for the preparation of a solution), sublingual tablets Liquids: drops, gels, juices, solutions, syrups, suspensions
Potentially critical	Regular release – drug undissolved	Coated tablets, film-coated tablets, hard capsules, (not) coated tablets
Critical	Modified release – sustained drug release/indigestible formulation	Enteric coated capsules/tablets, matrix tablets/capsules, prolonged-release capsules and tablets, softgel capsules

### Data analysis

The results are presented as absolute and relative frequencies in relation to the total number of patients and/or the total number of prescribed drugs. Due to the limited number of patients, we focused on descriptive statistics and did not perform any further statistical tests. All the data were recorded and analysed via Microsoft Excel 2019 (Microsoft 365) software. To obtain the corresponding ATC code for each drug prescription, the official version of the ATC index of the Scientific Institute of the AOK was used. Data are presented as median and Q25/Q75 (25%-/75%-quartile), as appropriate.

## Results

### Patient characteristics

85 patients fulfilling the inclusion criteria were enrolled in the patient chart review analysis, of whom 68 (80.0%) were inpatients and 17 (20.0%) were outpatients. The patient cohort comprised 37 females (43.5%) and 48 males (56.5%). The median age of the patients was 61 years (min–max. 21–88; Q25: 50; Q75: 71). A total of 598 drugs were recorded, comprising 147 distinct active ingredients. The stoma types were categorized as follows: 42.4% ileostomy, 2.3% jejunostomy, and 55.3% colostomy (Table 2).

**Table 2:** Patient characteristics for the patient chart review

			Median	(Q25; Q75)	Spread (min–max)
Total		**85**			
	Outpatient 68	Inpatient 17			
Age [y]			61	(50; 71)	21–88
Drugs		598	7	(4; 10)	0–17
Sex		**Female**		48 (56.5%)	
		**Male**		37 (43.5%)	
Stoma Location		**Colostomy**		47 (55.3%)	
		**Ileostomy**		36 (42.4%)	
		**Jejunostomy**		2 (2.3%)	
Diagnosis		**Colorectal Carcinoma**		36 (42.4%)	
		**Diverticulitis**		12 (14.1%)	
		**Chronic Inflammatory Bowel Disease**		8 (9.4%)	
		**Other neoplasms (e.g. ovarian, peritoneal carcinomatosis etc.)**		16 (18.8%)	
		further diagnoses*		13 (15.3%)	

*Conglomerate tumor of the ileocecal region, perineal acne inversa, gluteal abscess, rectovaginal fistula, conglomerate tumor of the sigmoid colon, abscesses and fistulas, colonic ischaemia, chronic colonic ileus, fistula of the stomach into the transverse colon and multiple intraenteric abscesses, Ogilvie syndrome, peritonitis, complex anal fistula, faecal incontinence, Hirschsprung's disease

Among those 85 patients included in the chart review analysis, 50 (58.8%) agreed to participate in the patient interview. In 28 of the patients, the post-operative period was less than a fortnight ago; these were excluded in accordance with the exclusion criteria. Seven patients declined to participate in the interview phase of the study. It is noteworthy that all patients in the interview phase had previously undergone a chart review (Table 3).

**Table 3:** Patient characteristics for the patient interview

		Median	(Q25; Q75)	Spread (min–max)
Total	**50**			
Age [y]		61	(49; 72)	21–88
Time after Surgery [m]		13	(4; 42)	2–429
Sex	**Female**		25 (50%)	
	**Male**		25 (50%)	
Stoma Type	**Colostomy**		25 (50%)	
	**Ileostomy**		25 (50%)	

### Patient chart review (i)

In order to characterise the groups of perorally administered drugs, they were categorised in Table 4 according to the first level of the ATC classification and refer to all orally administered drugs identified during the evaluation of charts and interviews.

**Table 4:** Drug categorizing according to ATC classification (*n* = 147)

ATC	Classification	*n*	**%**
A	Alimentary system and metabolism	**40**	27.2
B	Blood and hematopoietic organs	**12**	8.2
C	Cardiovascular system	**29**	19.7
D	Dermatics	-	-
G	Urogenital system and sex hormones	3	2.0
H	Systemic hormone, excluding sex hormones and insulins	7	4.8
J	Anti-invasive agents for systemic use	1	0.7
L	Antineoplastic and immunomodulatory agents	3	2.0
M	Musculoskeletal system	4	2.7
N	Nervous System	**41**	27.9
P	Antiparasitics, insecticides and repellents	-	-
R	Respiratory system	-	-
S	Senses	-	-
V	Varia	7	4.8

Major representatives of ATC group N were metamizole sodium, hydromorphone, zopiclone and pregabalin. Pantoprazole, macrogol (combinations) and cholecalciferol were important representatives of group A. ATC group C included drugs such as torasemide, ramipril and atorvastatin. Finally, ATC group B with antithrombotics such as acetylsalicylic acid was being particularly common. The classification of perorally prescribed drugs on the basis of the pharmaceutical formulation revealed that 55% (332/598) were normal-release drugs. The percentages of modified-release and accelerated-release formulations were 22.1% (132/598) and 22.4% (134/598), respectively.

### Patient interview (ii)

#### Undissolved drugs in the stoma bag (ii.a)

The majority of the interviewed patients (39/50; 78.0%) indicated that they had not encountered any instances of undissolved drugs in their stoma bag. A total of 11/50 (22%) of the respondents reported having experienced at least one instance. Among those patients, 8 out of 11 had ileostomy, and 3 had colostomy. A further 82% (9 patients) of those 11 who discovered undissolved residues in the stoma bag were able to identify which drug was of interest. An overview of the drugs found is shown in Table 5.

**Table 5:** Overview of the unexpected drugs found in stoma bags by the patients

Drug (Brand)	Active ingredient	Pharmaceutical formulation	Alternative formulation (optional)
Loperamid, generic	Loperamide	tablets (potentially critical)	Drops
Renvela®	Sevelamer	film-coated tablet (potentially critical)	Suspension
Novamin, generic	Metamizol-Natrium	film-coated tablet (potentially critical)	Drops
Pantoprazol, generic	Pantoprazole	enteric-coated tablet (critical)	Esomeprazole capsules or MUPS tablets, water dispensable pellets
Venlafaxin	Venlafaxin	enteric coated tablet (critical)	capsules, water dispensable pellets
Kreon®	Multienzymes (lipase, protease etc.)	capsule, enteric coated pellets (critical)	water dispensable pellets
Hydromorphon, generic	Hydromorphone	sustained tablet (critical)	capsules, water dispensable pellets or TTS (patches)
Tamsulosin, generic	Tamsulosin	sustained tablet (critical)	capsules, water dispensable pellets
Elontril®	Bupropion	sustained capsule (critical)	none, therapy change necessary

Overall, 7% (3/50) of the patients had short bowel syndrome, while 10% (5/50) had a chronic inflammatory bowel disease. None of the patients had a high-output stoma. Among 12 patients, who had already found drugs in their ostomy bag, 9 had undergone a small bowel area resection.

#### Changes in drug efficacy and therapy (ii.b)

A subsequent analysis of all reported bag findings revealed that 7 out of 50 patients (14%) reported alterations in the therapeutic effect of the perorally administered drugs. Those patients were additionally requested to identify any specific drugs to be responsible for the efficacy changes. The reported events included inadequate pain treatment despite analgesic administration, insufficient therapeutic effect when antibiotics were prescribed, and observed fluctuations in blood pressure when antihypertensive medications were administered. A total of 6% (3/50) of the respondents did not answer this question, whereas 12% (6/50; 10% ileostomy patients and 2% colostomy patients) reported noticing changes in their therapy regimen. Once patients informed their physician about the presence of the undissolved drug within the bag, and it was probably not to be expected due to the galenics, the drug in question was typically modified (Table 5).

#### Patient information status (ii.c)

Concerning the information regarding alterations in drug efficacy resulting from the implementation of a stoma, 62.0% of the surveyed respondents (31/50) indicated that they had not been made aware of this possibility. A further 16.0% (8/50) could not recall the information while 22.0% (11/50) stated that they had been well informed.

## Discussion

The accelerated transit time in ostomy patients can be assumed to impair drug release, absorption, and consequently therapeutic efficacy. Therefore, the aim of this study was to identify unexpected, undissolved drugs in stoma bags. The purpose was to establish a logical relation with the pharmaceutical formulations used, classifying them into non-critical, potentially critical, and critical. The patient interviews revealed that approximately 20% of patients (10/50) had already experienced the unexpected presence of drugs in their stoma bags at least once (no known case of expected drug indigestion). Furthermore, 12% (6/50) reported that the insertion of the stoma resulted in a perceived alteration in drug therapy.

The absorption process is predominantly facilitated in the jejunum and the upper segments of the ileum. Consequently, patients who have undergone a colostomy generally experience fewer restrictions on absorption in comparison to those who have undergone an ileostomy [4, 5].

The survey indicated that both patients with ileostomy and those with colostomy could be affected by the presence of undissolved drugs in the stoma bag. This presence may be a consequence of drug release-related problems, unless it is expected due to the galenic properties of the formulation. Patients with ileostomy are subject to this phenomenon more frequently than those with colostomy. This is an understandable occurrence, given that the absorptive surface of patients with a colostomy often remains intact within the small intestine. However, neglecting the possibility of absorption issues in patients with a colostomy would be erroneous. As an example, a patient with a colostomy reported discovering drugs in the stoma bag on at least one occasion, who had additionally undergone small bowel resections and was diagnosed with Crohn's disease. Both are factors that can affect the absorption of drugs.

In contrast to patients who have undergone bariatric surgery, for whom there is already some evidence regarding the alteration in the efficacy of perorally administered drugs [14], such studies remain pending in ostomy patients. The described changes in the efficacy of analgesics, antihypertensives, and antibiotics in this study are undoubtedly feasible. Consequently, the efficacy of the treatment should be evaluated meticulously prior to changes in the therapeutic regimen. Definitive evidence of reduced efficacy would be feasible only through direct observation (e.g., therapeutic drug monitoring) or indirect (e.g., blood pressure) measurement in a larger study population and could be subject to future investigations.

However, a change in drug efficiency does not necessarily correlate with findings in the stoma bag. For example, the preparation may be entirely dissolved, yet owing to a significantly short transit time, not all of the active ingredients may have been absorbed. In the present study, a comparable example could be found with the drug bupropion and venlafaxine. For example, venlafaxine ER (extended release) is usually formulated as a pellet-filled capsule. These pellets consist of an inactive carrier core encased in a controlled release membrane. The capsule itself dissolves in the stomach, while the pellets release the active ingredient over several hours. These pellets could be found in the stoma bag. Bupropion XL (extended release) as another example contains the active ingredient in a hydrophilic or hydrophobic matrix that releases the active ingredient slowly. The extended release formulation has a longer release time, often over 24 h. The tablet shell or the insoluble matrix often remains visible in the stool and therefore also in the ostomy bag.

With venlafaxine ER and bupropion XL, matrix residues may remain in the stool. This is usually not a problem, but in ostomy patients, the absorption of the active substance may also be impaired. The no digestible nature of these formulations can result in misinterpretations, both in the context of routine clinical practice and in everyday patient lives.

A patient who is adequately educated and aware of their particular situation is best suited to contribute to the safe independent administration of drugs. In accordance with the established definition of van der Linde et al. [13], all identified formulations were classified as either critical or potentially critical. Consequently, the classification of pharmaceutical formulations can be used as a basis for the prescription of oral drugs, with the aim of reducing drug release-related problems in ostomy patients. Therefore, medication management including pharmaceutical prescription review is particularly important for ostomy patients and requires the special attention of an interprofessional treatment team consisting of physicians, nurses and pharmacists [12, 15–17]. It is particularly important for pharmacists to be aware of contraindicated drugs, special formulations and those that may result in alterations to the color or odor of excreta [14]. Nevertheless, it is highly recommended that pharmacists also be involved in the assessment of pharmaceutical formulations and their galenic properties as educational advice to prevent further drug release-related problems [12]. Supported by the advancement of knowledge of drug design and gastrointestinal pathophysiology, it may be feasible to accurately predict optimal absorption in the future, e.g., using computer modelling [12].

### Limitations

It could be assumed that recall bias will be present in the responses given during the interview, given that the events in question occurred in the past. The study relies on self-reports and perceptions of drug efficacy, both of which can be biased. A more comprehensive set of data concerning the changes in drug therapy was not collected as part of this study, since the physicians treating the patients were not interviewed. Furthermore, the study's one-sided patient population may not represent patients overall, so the findings may not be generalizable.

## Conclusion

A number of pharmaceutical formulations have been identified as having the potential to cause drug-related problems for patients with an ostomy. Approximately one in four to five patients frequently reported (part of) their drugs found in their bags. Patients’ lack of education highlights the need for a strategy to increase patient information status.

## Supplementary Information

Below is the link to the electronic supplementary material.

Supplementary file1 (DOCX 29 KB)

## Data Availability

The data sets analysed during the current study are not publicly available but are available from the corresponding author upon reasonable request.
